# The Effectiveness of Instant Messaging‐Based Interventions on Health Behavior Change: A Systematic Review and Meta‐Analysis

**DOI:** 10.1111/wvn.70066

**Published:** 2025-07-27

**Authors:** Tianxue Hou, Mu‐Hsing Ho, Huangqianyu Li, Chia‐Chin Lin

**Affiliations:** ^1^ School of Nursing Li Ka Shing Faculty of Medicine, The University of Hong Kong Hong Kong SAR China; ^2^ Alice Ho Miu Ling Nethersole Charity Foundation Hong Kong SAR China; ^3^ Research Unit for Enhancing Well‐Being in Vulnerable and Chronic Illness Populations, Faculty of Nursing Chulalongkorn University Bangkok Thailand

**Keywords:** behavior change, health behavior, instant message, intervention

## Abstract

**Background:**

Instant messaging‐based applications are increasingly used to deliver interventions designed to promote health behavior change. However, the effectiveness of these interventions has not been evaluated.

**Aims:**

This systematic review and meta‐analysis aimed to evaluate the effectiveness of instant messaging‐based interventions on health behavior change, addressing a gap in the literature regarding the impact of instant messaging on various health behaviors.

**Methods:**

We conducted comprehensive searches of six electronic databases (PubMed, EMBASE, Cochrane Library, PsycINFO, CINAHL Plus, and Web of Science) from their inception until July 2024, utilizing terms related to health behavior and instant messaging. Two authors independently screened studies and extracted data. Randomized controlled trials published in English that investigated the effects of instant messaging‐based interventions on health behavior change, including physical activity, sedentary behavior, sleep, diet/nutrition, cancer screening, smoking cessation, and alcohol consumption were included. We used the revised Cochrane Risk‐of‐Bias Tool to assess the quality of the studies.

**Results:**

Fifty‐seven randomized controlled trials published between 2014 and 2024 were included. The results showed that compared with the control groups, instant messaging‐based interventions had statistically significant differences in physical activity (SMD = 0.52, 95% CI [0.21, 0.83], *p* < 0.001) and sleep (SMD = −0.93, 95% CI [−1.44, −0.42], *p* < 0.001). It also significantly impacted smoking cessation (OR = 1.88, 95% CI [1.28, 2.7], *p* < 0.001). However, it did not influence sedentary behavior (SMD = 0.25, 95% CI [−0.24, 0.74], *p* = 0.01) or diet/nutrition (SMD = 0.01, 95% CI [−0.31, 0.34], *p* < 0.001).

**Linking Evidence to Action:**

Instant messaging‐based interventions are promising in enhancing health behavior change, including physical activity, sleep, and smoking cessation. Leveraging real‐time communication and multimedia content can improve patient engagement and intervention effectiveness.

## 1 Introduction

Non‐communicable diseases (NCDs) are the leading cause of global mortality and morbidity (Global Burden of Disease Consortium 2020). According to the World Health Organization (WHO), NCDs account for approximately 71% of all deaths worldwide, equaling about 41 million people annually (Global Burden of Disease Consortium 2020). Unhealthy lifestyles, such as lack of physical activity, smoking, and excessive alcohol consumption, are the primary causes of NCDs (Lim et al. 2012). This highlights the urgent need for public health interventions to promote health behavior change.

Traditional methods for promoting health behavior change include educational campaigns, individual counseling, and group program interventions (Bull et al. 2014; Conn et al. 2011). These approaches focus on increasing awareness, enhancing self‐efficacy, and fostering behavioral intentions (Shimazaki et al. 2022). However, they are often subject to limitations; for example, though educational campaigns can raise awareness about health risks, they may not effectively lead to behavior change due to a lack of engagement or personal relevance to the target individual (Rigg et al. 2014). Additionally, a one‐size‐fits‐all approach often fails to address the diverse needs and motivations of different populations (Rigg et al. 2014). Many traditional interventions are time‐limited, expensive, and lack ongoing support that is crucial for sustaining long‐term behavior change (Iribarren et al. 2021). As public health interventions evolve, innovative approaches that integrate technology are needed to address diverse population needs and provide ongoing support to enhance the effectiveness of health behavior change interventions. Therefore, integrating remote mobile health into health behavior change strategies has emerged as a promising avenue.

Instant messaging is a form of online communication that allows real‐time communication through platforms like WhatsApp, WeChat, and Facebook Messenger, facilitating immediate exchange of text, images, audio, and video messages (Li, Ho, et al. 2022). Instant messaging applications support one‐on‐one and group conversations and file sharing, enhancing connectivity across geographical boundaries (Chiang et al. 2021).

Recently, the use of instant messaging in health interventions has become more prevalent, primarily driven by its ability to facilitate real‐time communication and provide personalized support. Various studies highlight the effectiveness of instant messaging‐based interventions in managing health behavior (Alley et al. 2016; Chau et al. 2024; Wang et al. 2019). For instance, instant messaging applications enable healthcare providers to deliver brief motivational interviews, helping cancer patients adopt and maintain regular physical activity (Cheung et al. 2022). The WHO has recognized the potential of such technologies in promoting health and enhancing treatment compliance, especially when traditional face‐to‐face interactions are limited, such as during the COVID‐19 pandemic (Kay et al. 2011). Despite the widespread use of instant messaging platforms and their potential to reach large populations in a cost‐effective manner, there is a notable lack of reviews evaluating their effectiveness. This gap is particularly significant given the rapid adoption of instant messaging applications in everyday communication. Therefore, there is a need for a systematic review and meta‐analysis that examines the effectiveness of instant messaging interventions in health behavior change, addressing these gaps and providing evidence‐based recommendations for future practice and research.

This is the first systematic review and meta‐analysis to explore the effectiveness of instant messaging‐based interventions on health behavior change. This study aimed to systematically appraise and examine the effectiveness of instant messaging on health behavior change.

## 2 Methods

The systematic review and meta‐analysis adhered to the Preferred Reporting Items for Systematic Reviews and Meta‐Analyses guidelines and was registered in the International Prospective Register of Systematic Reviews (PROSPERO registration number: CRD42024579283).

### 2.1 Search Strategy

We conducted comprehensive searches of six electronic databases (MEDLINE via PubMed, EMBASE, Cochrane Library, PsycINFO, CINAHL Plus, and Web of Science Core Collection) from their inception until July 2024. The search utilized terms and relevant medical subject headings related to health behavior (e.g., physical activity, sedentary behavior, sleep, diet/nutrition, cancer screening, smoking cessation, and alcohol consumption) and instant messaging. An example of a search strategy for MEDLINE is available in Table S1.

### 2.2 Eligibility Criteria

The eligibility criteria were defined using the Population, Intervention, Comparison, Outcomes, and Study Design framework. We included randomized controlled trials (RCTs) with no population restrictions, examining instant messaging‐based interventions (e.g., WeChat, WhatsApp, and Skype) delivered in any setting (e.g., clinical, community, or home environments) without constraints on intervention duration or intensity. Eligible comparators encompassed both passive controls (e.g., usual care) and active controls (e.g., alternative interventions). Primary outcomes focused on behavioral measures across seven domains: (1) physical activity (measured by metabolic equivalents [MET], moderate‐to‐vigorous physical activity [MVPA] duration, and 6‐min walk test [6MWT]); (2) sedentary time (minutes/day); (3) sleep quality metrics; (4) cancer screening adherence rates; (5) nutritional status (assessed using glycated hemoglobin [HbA1c] levels, hemoglobin concentrations, dietary energy intake, and body mass index); (6) smoking cessation outcomes (biochemically verified abstinence preferred); and (7) alcohol consumption patterns. Exclusion criteria applied to non‐RCT designs, conference abstracts, review articles, theses, study protocols, and publications in languages other than English.

### 2.3 Study Selection and Data Extraction

Two reviewers independently screened titles and abstracts. Full‐text articles were reviewed independently by two reviewers, with any disagreements resolved through discussion with a third reviewer. Data extraction was performed by one reviewer and double‐checked by a second reviewer. Extracted information included (1) study characteristics: author, publication date, country, and design; (2) participant characteristics: sample size, age, and gender; and (3) intervention characteristics: instant messaging app usage, underpinning theories, duration, and retention rate.

### 2.4 Bias Assessments and Certainty of the Evidence

Two reviewers independently assessed the risk of bias using the revised Cochrane Risk of Bias tool 2.0 (Sterne et al. 2019). It assesses five domains: (1) the randomization process, (2) deviations from intended interventions, (3) missing outcome data, (4) outcome measurement, and (5) selection of reported results. Each study was rated as having a “low,” “some concerns,” or “high” risk of bias in overall judgment. The Grading of Recommendations Assessment, Development, and Evaluation system was used to evaluate the quality of evidence (Schunemann et al. 2008). The level of quality was rated as high, moderate, low, or very low. Any disagreement was resolved by a third reviewer.

### 2.5 Data Synthesis and Statistical Analyses

Separate meta‐analyses were conducted for each type of health behavior to estimate mean effects. Cluster‐Randomized Control Trials (CRCTs) were excluded from the meta‐analysis to prevent issues with a unit of analysis. A narrative synthesis was provided for excluded CRCTs. For missing data, we attempted statistical conversion or contacted authors for datasets. Studies were excluded listwise if neither approach succeeded.

The meta‐analysis included results with available post‐intervention values. Effect sizes were determined using data from the last follow‐up time point. For continuous data, we extracted participant numbers, mean scores, and standard deviations to calculate the standardized mean difference (SMD). SMD was interpreted based on Cohen's guidelines: small (*d* < 0.4), medium (*d* = 0.4–0.7), and large (*d* ≥ 0.70) (Cohen 2013). For dichotomous data, the number of individuals in each category was extracted to calculate odds ratios (OR). Heterogeneity was assessed using the *I* ^2^ statistic. *I* ^2^ values ranging from 0% to 40%, 30% to 60%, 50% to 90%, and 75% to 100% indicate unimportant, moderate, substantial, and considerable heterogeneity, respectively (Chandler et al. 2019). Given the variability of complex interventions across different studies, we employed a random‐effects model, which accounts for variations in effect sizes and allows for more generalizable and realistic conclusions (Borenstein et al. 2010). Due to the limited number of studies included for each type of health behavior, we only performed a Leave‐One‐Out analysis, using a Galbraith plot to assess heterogeneity, and conducted a publication bias assessment using Begg's rank test and Egger's regression test for smoking cessation, as it includes more than 10 articles (Bax et al. 2009). All analyses were conducted using Stata MP version 17 software.

## 3 Results

### 3.1 Study Selection

The initial search of the databases yielded 2135 articles, of which 1084 were considered potentially relevant after removing duplicates. A full‐text review was conducted for 85 articles. Eventually, 52 RCTs and 5 CRCTs were included in the final review (Figure S1).

### 3.2 Study Characteristics

Study characteristics are shown in Table 1. The articles were published between 2014 and 2024. There were 25 RCTs conducted in mainland China, 17 in Hong Kong, 3 in Saudi Arabia, 2 in the USA (Klaren et al. 2014; Valle et al. 2023), 2 in Turkey (Balmumcu and Unsal Atan 2021; Durmaz et al. 2019), 2 in Iran (Ghasemian et al. 2024; Shokri et al. 2023), 2 in Oman (Al‐Ghafri et al. 2020; Alghafri et al. 2018), 1 in Germany (Gieselmann and Pietrowsky 2019), 1 in Australia (Alley et al. 2016), 1 in Malaysia (Ahmad et al. 2018), and 1 in Thailand (Rojnawee et al. 2023). The review included 19,365 participants, and the sample sizes ranged between 33 and 2000 (Kwan et al. 2020; Tang et al. 2023). The participants' average age ranged from 3.3 years to 70 years (Zhang et al. 2022, 2021). The intervention duration ranged from 21 days to 12 months (Alghafri et al. 2018; Li, Wong, et al. 2022). Retention rates ranged between 38% and 96% (Alley et al. 2016; Xu et al. 2020). Most interventions targeted smoking cessation (*n* = 21), while 14 studies targeted physical activity, 12 targeted diet and nutrition, 5 targeted sleep quality, 3 targeted sedentary behavior, 1 targeted cancer screening, and 1 targeted alcohol consumption. Nearly half of included studies (*n* = 27) based their interventions on at least one theoretical model, including the following: social cognitive theory (*n* = 7), transtheoretical model (*n* = 7), theory of planned behavior (*n* = 2), behavioral change theory (*n* = 2), transforming management model (*n* = 2), award model (*n* = 2), behavior change wheel model (*n* = 2), behavioral theory, social determinants of health, socioecological model of health behavior, communication theory, health action process approach model, cognitive behavioral theory, acceptance, and commitment therapy model, and behavioral change technique (Table 1).

**TABLE 1:** Characteristics of the included studies (*n* = 57).

Author	Year	Country/Region	Sample	Age	Male	App	Theoretical model	Duration	Retention (%)	Design	Target behavior
Alshahrani	2021	Saudi Arabia	103	—	0.0%	WhatsApp	—	10 weeks	93.6	RCT	Physical activity
Lin	2021	China	60	IG: 66.16 ± 6.69 CP: 67.26 ± 7.99	2.0%	WeChat	—	6 months	—	RCT	Physical activity
Bi	2021	China	200	IG: 69.42 ± 7.48 CP: 68.87 ± 5.33	66.5%	WeChat	—	12 weeks	—	RCT	Physical activity
Shokri	2024	Iran	41	50–75	—	WhatsApp	—	3 months	—	RCT	Physical activity
Cheung	2024	Hong Kong	161	12.4 ± 2.4	57.8%	WhatsApp	—	6 months	IG: 92.6 CP: 88.6	RCT	Physical activity
Su	2021	Hong Kong	146	IG: 55.53 ± 7.30 CP: 56.03 ± 7.02	IG: 84.9% CP: 82.2%	WeChat	Social cognitive theory	—	90	RCT	Physical activity
Xu	2020	China	76	23–67	55.3%	WeChat	Behavioral theory	6 months	96	RCT	Physical activity
Alley	2016	Australia	154	54	24.0%	Skype, Google and so on	Theory of planned behavior and communication theory	2 months	38	RCT	Physical activity
Wu	2023	China	1610	6–20 months	5.9%	WeChat	—	1 months	—	CRCT	Physical activity
Kwan	2020	Hong Kong	33	71	15.0%	WhatsApp	—	3 months	91	Pilot RCT	Physical activity
Al‐Ghafri	2019	Oman	232	44.2 ± 8.1	4.9%	WhatsApp	—	—	75	CRCT	Physical activity
Valle	2022	USA	280	33.40 ± 4.80	18.2%	Facebook	Social cognitive theory	6 months	90	RCT	Physical activity
Peng	2017	China	98	66.3 ± 1.5	59.2%	WeChat	—	2 months	84.7	RCT	Physical activity
Chan	2022	Hong Kong	139	IG: 59.8 ± 6.4 CP: 59.8 ± 6.8	IG: 62.9% CP: 72.7%	WhatsApp	Health action process approach model	3 months	81.3	Pilot RCT	Physical activity
Saquib	2023	Saudi Arabia	207	22.6 ± 1.3	0.0%	WhatsApp	Social cognitive theory	3 months	—	RCT	Sedentary behavior
Li	2023	China	42	23.59 ± 1.39	37.0%	WeChat	Behavioral changing theory	1 month	—	RCT	Sedentary behavior
Klaren	2014	USA	70	IG: 49.4 ± 9.2 CP: 0.3 ± 9.1	IG: 27% CP: 18%	Skype	Social cognitive theory	6 months	—	pilot RCT	Sedentary behavior
Gieselmann	2019	German	73	Face to Face: 39.30 ± 14.47 Chat: 39.74 ± 11.16 Waiting list: 42.74 ± 11.73	Face to Face group: 52% Chat group: 44% Waiting list: 47%	Skype	—	—	—	RCT	Sleep
Han	2021	China	132	IG: 44.4 ± 8.2 CP: 45.5 ± 7.9	0.0%	WeChat	—	3 months	—	RCT	Sleep
Wang	2023	China	175	IG: 5.90 ± 14.21 CP: 5.70 ± 14.31	IG: 53.4% CP: 54	WeChat	Transforming management model	—	—	RCT	Sleep
Duan	2023	China	146	IG: 4.0 ± 2.0–12.0 CP: 4.0 ± 1.0–11.0	IG: 69.4% CP: 59.5%	WeChat	—	6 months	—	RCT	Sleep
Li	2022	Hong Kong	333	IG: 41.67 ± 13.57 CP: 42.59 ± 13.08	IG: 24% CP: 16.2%	WhatsApp	—	21 days	72.9–83.3	RCT	Sleep
Ye	2024	China	174	IG: 57 ± 3.8 CP: 57 ± 3.8	IG: 53% CP: 58%	WeChat	—	—	—	RCT	Diet/nutrition
Wang	2022	China	56	IG: 69.07 ± 6.53 CP: 67.50 ± 9.34	IG: 5.0% CP: 6.7%	WeChat	—	6 months	—	RCT	Diet/nutrition
Al‐Hamdan	2021	Saudi Arabia	253	Education program: 42.9 ± 12.2 WhatsApp education program: 43.7 ± 8.1 CP: 5.9 ± 7.1	0.0%	WhatsApp	—	3 months	—	RCT	Diet/nutrition
Kang	2021	China	180	—	63.3%	WeChat	—	3 months	88.9	RCT	Diet/nutrition
Lin	2021	China	102	IG: 4.2 ± 3.6 CP: 4.3 ± 3.9	—	WeChat	—	—	—	RCT	Diet/nutrition
Liu	2024	China	153	32.7 ± 7.5	36.6%	WeChat	—	—	—	RCT	Diet/nutrition
Chen	2023	China	102	36.76 ± 4.84	0.0%	WeChat	Social cognitive theory and social determinants of health	6 months	85	Pilot RCT	Diet/nutrition
Zhang	2022	China	84	IG: 3.3 ± 3.1 CP: 3.6 ± 3.3	IG: 75% CP: 82.3%	WeChat	—	—	—	RCT	Diet/nutrition
Ahmad	2018	Malaysia	134	IG: 39.8 ± 3.6 CP: 41.3 ± 5.7	IG: 58.2% CP: 55.2%	WhatsApp and Facebook	Social cognitive theory	4 months	—	RCT	Diet/nutrition
Alghafri	2018	Oman	232	4.2 ± 8.1	4.9%	WhatsApp	Socioecological model of health behavior and the behavior change wheel model	12 months	75	CRCT	Diet/nutrition
Ding	2020	China	215	3.3 ± 2.8	0.0%	WeChat	—	—	—	RCT	Diet/nutrition
Xia	2022	China	156	6.04 ± 12.56	63.0%	WeChat	—	6 months	76.9	RCT	Diet/nutrition
Ghasemian	2024	Iran	400	IG: 39.45 ± 13.90 CP 39.89 ± 13.72	37.8%	WhatsApp	The theory of planned behavior	3 months	—	RCT	Cancer screening
Luo	2022	China	403	3.5 ± 9.6	88.9%	WeChat	—	—	54	RCT	Smoking cessation
Zhao	2021	Hong Kong	119	—	8.7%	WhatsApp	Behavior change model	2 months	68.9	RCT	Smoking cessation
Li	2024	Hong Kong	1254	42.1 ± 16.2	76.8%	WhatsApp	—	3 months	8.1	CRCT	Smoking cessation
Cheung	2015	Hong Kong	136	4.5 ± 9.9	76.5%	WhatsApp and Facebook	Transtheoretical model	2 months	86.8	CRCT	Smoking cessation
Chen	2020	China	80	25–44	10.0%	WeChat	Behavior Change Wheel framework	6 weeks	88.8	RCT	Smoking cessation
Durmaz	2019	Turkey	132	39.3 ± 12.1	6.6%	WeChat	—	3 months	IG: 59.1 CP: 76.1	RCT	Smoking cessation
Lin	2022	China	772	—	99.2%	WeChat	Transtheoretical model	3 months	—	RCT	Smoking cessation
Luo	2021	China	403	Urban: 3.3 ± 9.3 Suburban: 27.8 ± 6.5 Rural: 28.1 ± 8.5	88.8%	WeChat	Transtheoretical model	2 weeks	—	RCT	Smoking cessation
Tang	2023	China	2000	—	93.6%	WeChat	Cognitive behavioral theory	14 weeks	86.6	RCT	Smoking cessation
Weng	2023	Hong Kong	1166	—	IG: 77.5% CP: 8.6%	WhatsApp	AWARD model	3 months	71	RCT	Smoking cessation
Wang	2019	Hong Kong	1185	41.5 ± 14	77.0%	WhatsApp	AWARD model	3 months	77	CRCT	Smoking cessation
Weng	2021	Hong Kong	1131	—	92.0%	WhatsApp	Acceptance and Commitment Therapy Model	3 months	86.3	CRCT	Smoking cessation
Balmumcu	2021	Turkey	50	27.4 ± 6.1	0.0%	WhatsApp	Transtheoretical model	—	—	RCT	Smoking cessation
Guo	2023	Hong Kong	664	18–60+	74.4%	WhatsApp	Transtheoretical model	13 weeks	73.2	RCT	Smoking cessation
Luk	2023	Hong Kong	108	45.1 ± 1.9	75.0%	WhatsApp	—	3 months	85	Pilot RCT	Smoking cessation
Rojnawee	2023	Thailand	314	IG: 33.13 ± 15.62 CP: 34.07 ± 15.24	89.8%	—	Transtheoretical model	6 months	—	RCT	Smoking cessation
Zhao	2024	Hong Kong	884	18–60+	82.3%	WhatsApp or WeChat	Transtheoretical model	3 months	79.5	RCT	Smoking cessation
Li	2022	Hong Kong	60	IG: 44.3 ± 1.2 CP: 48.1 ± 12.0	IG: 83.3% CP: 86.7%	WhatsApp or WeChat	—	6 months	71.7	Pilot RCT	Smoking cessation
Wu	2024	Hong Kong	700	18–60+	IG: 7.6% CP: 73.7%	WhatsApp or WeChat	Social cognitive theory and the transtheoretical model	3 months	63.7	RCT	Smoking cessation
Wang	2021	China	221	45 ± 35–53	91.9%	WeChat	—	3 months	—	RCT	Smoking cessation
Zhang	2021	China	110	22–70	93.6%	WeChat	—	28 days	95.4	RCT	Smoking cessation
Chau	2024	Hong Kong	722	21.1 ± 3.5	47.8%	—	Behavioral change technique	3 months	85	RCT	Alcohol consumption

Abbreviations: CG, control group; CRCTs, cluster‐randomized control trials; IG, intervention group.

### 3.3 Meta‐Analyses Results

The summary of findings and certainty of evidence assessment for each outcome is summarized in Table S2. The synthesized results regarding physical activity, sedentary behavior, sleep, diet/nutrition, and smoking cessation are further described.

#### 3.3.1 Physical Activity

**FIGURE 1:**
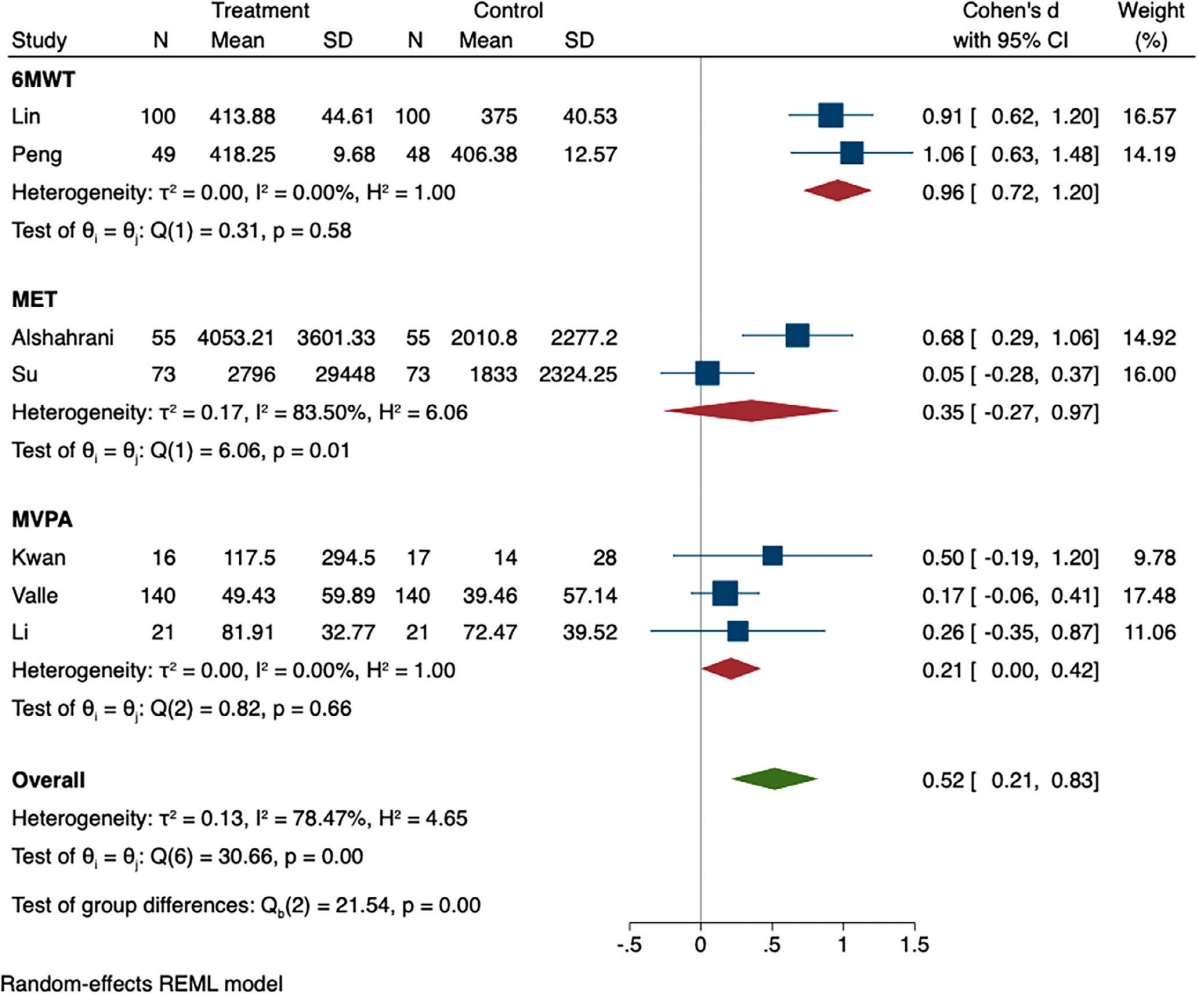
Forest plot of effect sizes of instant messaging‐based intervention on physical activity.

The synthesized effect size from seven studies demonstrated significant differences in total physical activity between intervention and control groups at post‐intervention (SMD = 0.52, 95% CI [0.21, 0.83], *p* < 0.001). The heterogeneity test showed significance among physical activity outcomes (*I* ^2^ = 78.47%, *p* < 0.001). For the subgroup analyses, 6MWT (SMD = 0.96, 95% CI [0.72, 1.20], *p* = 0.58) had statistically significant differences between the two groups. However, MET (SMD = 0.35, 95% CI [−0.27, 0.97], *p* = 0.01) and MVPA (SMD = 0.21, 95% CI [0.00, 0.42], *p* = 0.66) had no significance between the two groups, as shown in Figure 1.

#### 3.3.2 Sedentary Behavior

**FIGURE 2:**
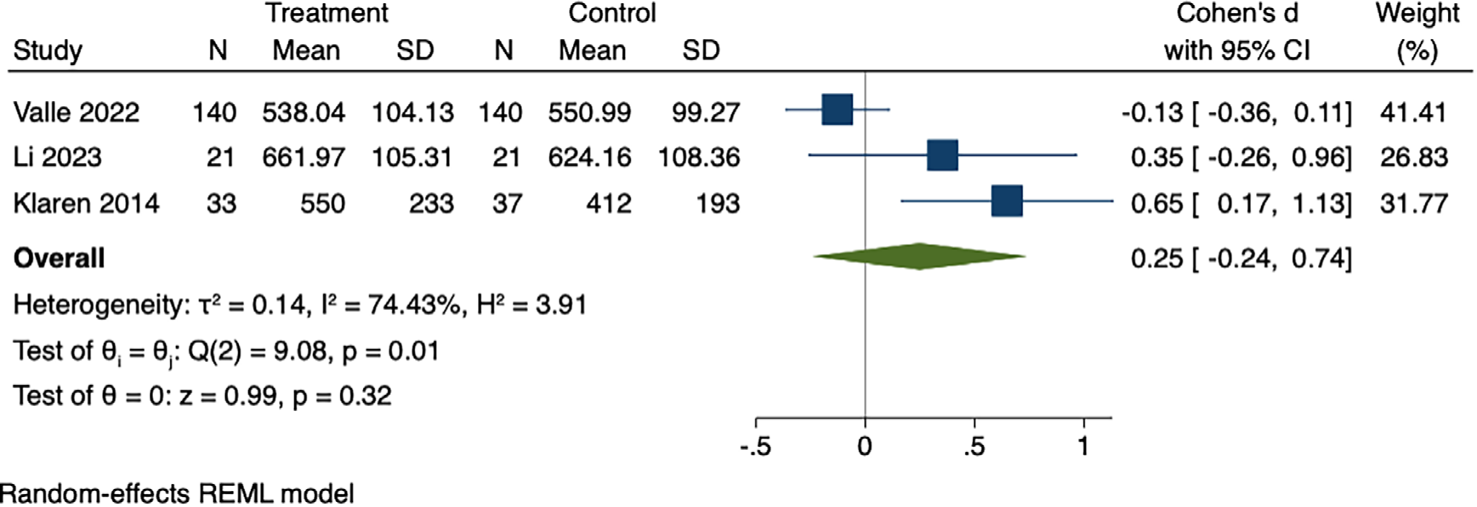
Forest plot of effect sizes of instant messaging‐based intervention on sedentary behavior.

There were only three studies examining sedentary behavior. Results did not show a significant difference in sedentary behavior between the two groups at postintervention (SMD = 0.25, 95% CI [−0.24, 0.74], *p* = 0.01). Significant heterogeneity was observed among sedentary behavior (*I* ^2^ = 74.43%, *p* < 0.001), as shown in Figure 2.

#### 3.3.3 Sleep

**FIGURE 3:**
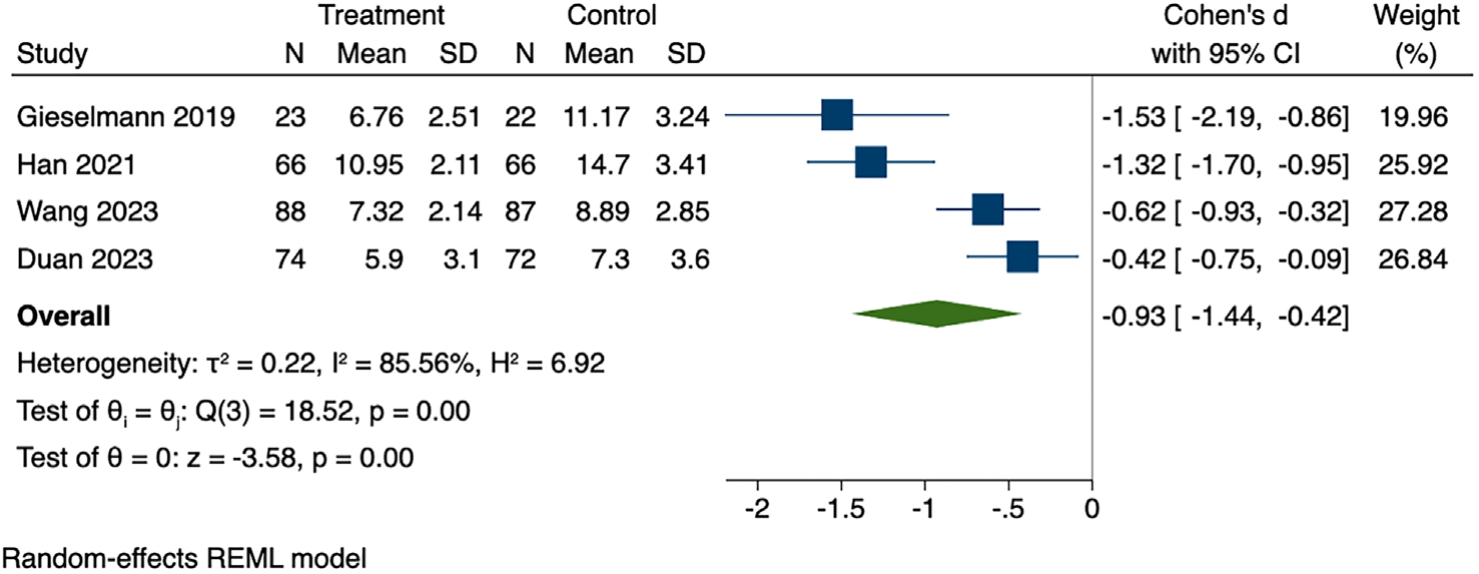
Forest plot of effect sizes of instant messaging‐based intervention on sleep.

Four studies examining health behavior related to sleep. Among these studies, significant differences in sleep quality were demonstrated between the two groups (SMD = −0.93, 95% CI [−1.44, −0.42], *p* < 0.001), as shown in Figure 3. Significant heterogeneity was observed among sleep behavior (*I* ^2^ = 85.56%, *p* = 0.01).

#### 3.3.4 Diet/Nutrition

**FIGURE 4:**
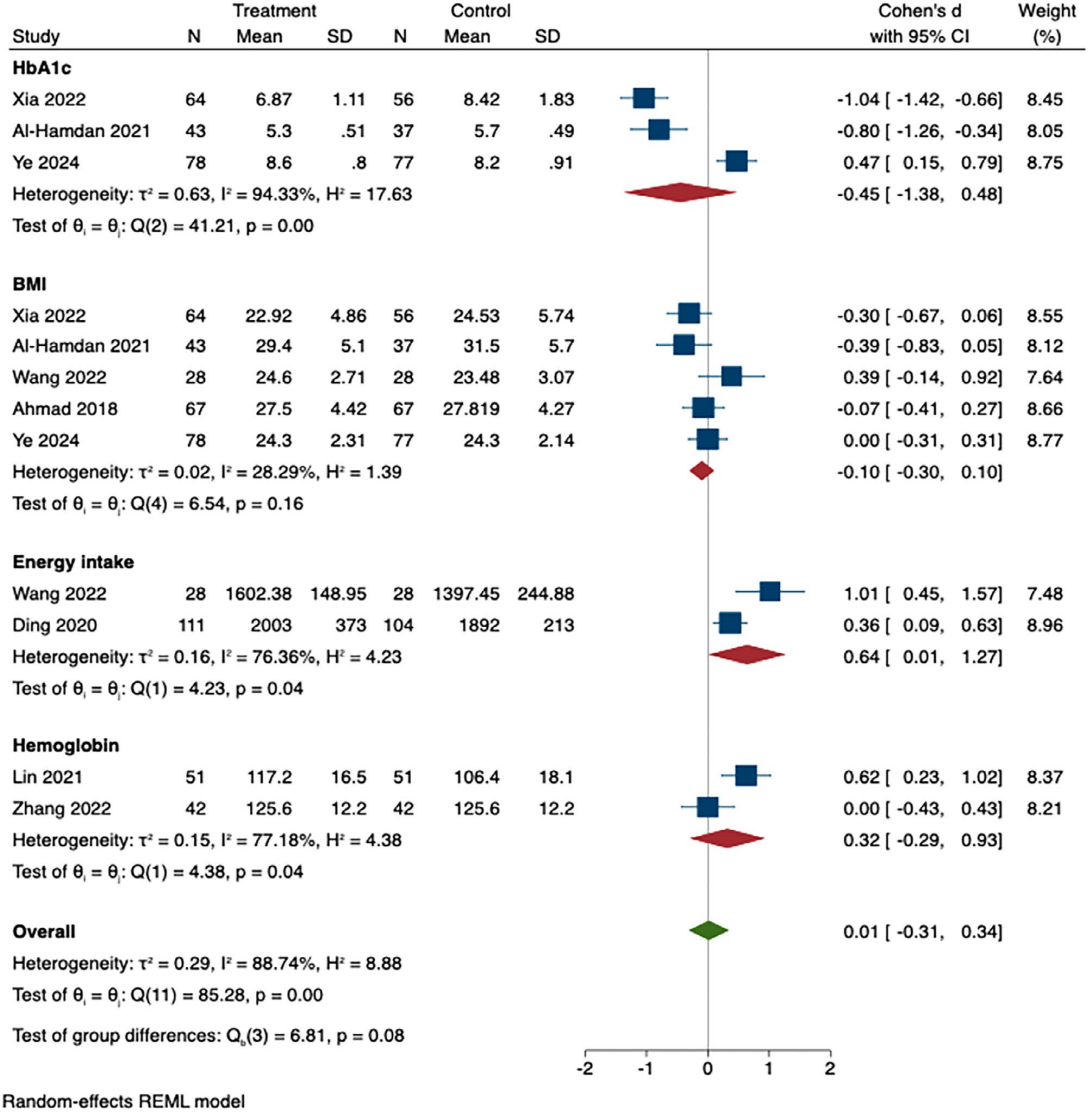
Forest plot of effect sizes of instant messaging‐based intervention on diet/nutrition.

Eight studies examined diet or nutrition‐related behavior change. The overall pooled SMD was 0.01 (95% CI [−0.31, 0.34], *p* < 0.001). There was substantial heterogeneity between the studies (*I* ^2^ = 9.34%, *p* < 0.001). For the subgroup analyses, energy intake (SMD = 0.64, 95% CI [0.01, 1.27], *p* = 0.04) had significant differences between the two groups. However, HbA1c (SMD = −0.45, 95% CI [−1.38, 0.48], *p* < 0.001), BMI (SMD = −0.1, 95% CI [−0.3, 0.1], *p* = 0.16) and hemoglobin (SMD = 0.32, 95% CI [−0.29, 0.93], *p* = 0.04) had no significant difference between the two groups, as shown in Figure 4.

#### 3.3.5 Smoking Cessation

**FIGURE 5:**
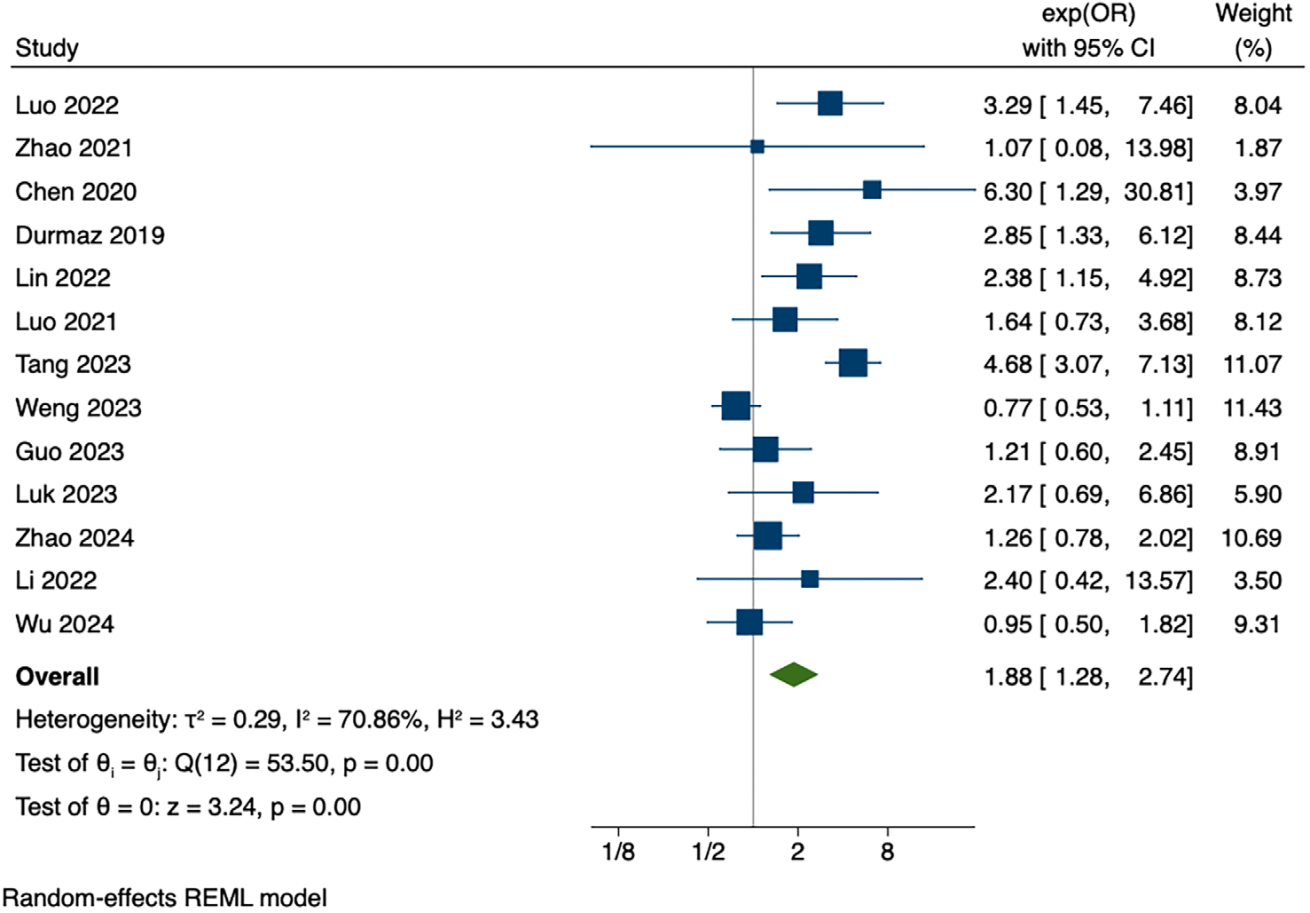
Forest plot of effect sizes of instant messaging‐based intervention on smoking cessation.

There was a significant effect for smoking cessation in a meta‐analysis of 13 studies (OR = 1.88, 95% CI [1.28, 2.7], *p* < 0.001), as shown in Figure 5.

### 3.4 Risk of Bias and Heterogeneity Assessments

The methodological quality assessment results were summarized in Supplement Material Table S3. Three studies were rated as low risk of bias, and 54 studies were classified as having some concerns. Most studies exhibited low risk in the domains of the randomization process, missing outcome data, and measurement of the outcome. However, some concerns were identified in the domains of deviations from the intended intervention and the selection of reported results. Specifically, 30 studies showed low risk across all domains except for some concerns in the selection of the reported results. Seven studies showed low risk across all domains. In contrast, four studies displayed some concerns in multiple domains, including deviations from the intended intervention and selection of the reported results (Table S3).

Due to the heterogeneity in the presented models, we conducted further analyses to test the robustness of the results. The Leave‐One‐Out analysis revealed that the effect sizes of Weng et al. (2023) and Wu et al. (2024) are statistically significantly higher than the overall effect size, indicating they may be potential outliers (Figure S2). Through the Galbraith plot, we found that the studies by Tang et al. (2023) and Weng et al. (2023) were close to the edges of the 95% CI regression line, suggesting that they might be the anomalies causing heterogeneity (Figure S3). After removing these three studies, the heterogeneity was significantly reduced, indicating that these studies were major sources of heterogeneity (OR = 1.94, 95% CI [1.41, 2.67], *p* < 0.001, *I* ^2^ = 23.82%).

### 3.5 Publication Bias

The assessment of publication bias revealed slight asymmetry in the funnel plot of smoking cessation studies. However, Begg's test (*p* = 0.30) and Egger's test (*p* = 0.45) indicated a low likelihood of publication bias in these studies, as shown in Figure S4.

## 4 Discussion

This is the first systematic review and meta‐analysis to comprehensively evaluate the effectiveness of instant messaging‐based interventions in changing health‐related behaviors. We identified 57 trials involving 19,365 participants, with findings suggesting that instant messaging‐based interventions are effective for health behavior change, though substantial heterogeneity was observed.

Small to large effect size improvements for health behaviors were observed, including physical activity, sleep, and smoking cessation, with SMDs ranging from −0.93 to 0.21. These SMDs suggest that instant messaging‐based interventions can substantially impact health outcomes. Comparatively, other meta‐analyses have reported smaller effect sizes for face‐to‐face and various digital, web‐based, or chatbot interventions, with SMDs between 0.13 and 0.68 (Bull et al. 2014; Champion et al. 2019; Conn et al. 2011; Singh et al. 2023). This comparison highlights the potential superiority of instant messaging interventions in promoting health behavior change. The larger effect sizes observed in instant messaging‐based interventions may be attributed to their real‐time, interactive nature, which offers immediate feedback and personalized support. For example, individuals participating in smoking cessation programs can benefit from real‐time peer support and shared experiences, which increase motivation and adherence to the intervention (Wang et al. 2019). In contrast, face‐to‐face and non‐digital interventions may lack the same level of immediacy and personalization. Other digital interventions that are truly interactive, such as chatbot interventions, may not offer the same level of human‐like engagement as instant messaging‐based interventions (Singh et al. 2023). These findings underscore the effectiveness of instant messaging applications as a powerful tool for health interventions, potentially offering more impactful results than other methods.

Our analysis found that instant messaging‐based interventions had no significant impact on reducing sedentary behavior. A previous study has demonstrated that internet interventions can be effective in promoting physical activity and reducing sedentary behavior, which is in contrast with our study (Afshin et al. 2016). The discrepancy between our findings and earlier studies may be due to differences in the types of interventions studied, the populations targeted, or the specific outcomes measured (Klaren et al. 2014; Li et al. 2023; Saquib et al. 2023). One potential reason for the lack of effect on sedentary behavior could be that the immediate feedback and support offered by instant messaging alone may not be sufficient to overcome the deeply ingrained habits and environmental factors that contribute to sedentary lifestyles (Azaiza et al. 2011). Additionally, the brevity and informality of instant messages may not provide the depth of engagement needed to promote sustained behavior change (Hall et al. 2015).

Similarly, our meta‐analysis found that instant messaging‐based interventions did not affect overall diet or nutrition‐related behavior change. Interestingly, while there was a statistically significant difference in energy intake between the intervention and control groups, other key indicators such as HbA1c, BMI, and hemoglobin did not show statistically significant differences. This finding is consistent with another review of digital interventions, which also reported positive effects on energy intake (Prowse and Carsley 2021). The discrepancy may be due to differences in the intensity, duration, and theoretical underpinnings of the interventions (Ahmad et al. 2018; Chen et al. 2023; Xia et al. 2022). The lack of significant impact on HbA1c, BMI, and hemoglobin levels could stem from two reasons. First, these outcomes may require longer‐term interventions to observe statistically significant changes. Second, dietary behavior is complex and influenced by a multitude of factors, including socioeconomic status, cultural norms, and individual preferences, which may not be adequately addressed by instant messaging interventions alone (Gherasim et al. 2020; Mora and Golden 2017).

About half of the included studies mentioned a specific theory or model underlying the intervention, and the most frequently used theories were social cognitive theory (*n* = 7) and transtheoretical model (*n* = 7). To enhance the effectiveness of instant messaging interventions, researchers need to align their theoretical frameworks with specific health behavior goals. This alignment will not only optimize resources but also ensure that interventions are evidence‐based and tailored to the diverse needs of populations. As the use of instant messaging continues to expand, leveraging these theoretical models effectively can lead to more robust and impactful health behavior change interventions.

### 4.1 Implications for Future Research

This study provides evidence for the efficacy of using instant messaging‐based intervention in health behavior change. Instant messaging platforms such as WhatsApp and WeChat offer significant advantages over traditional short‐message services by enabling real‐time communication and the exchange of multimedia content, which enhances the effectiveness of health interventions (Chau et al. 2024). For instance, these platforms can send personalized exercise videos, dietary tips, and medication reminders that are more engaging and easier to understand than static text messages (Wang et al. 2019). This immediacy and interactivity are particularly beneficial in interventions requiring timely responses, such as managing chronic conditions like diabetes or hypertension.

Future research needs to provide more detailed descriptions of the message design (Lustria et al. 2013). Detailed intervention descriptions can improve the development and customization of messages to enhance engagement, relevance, and impact. Additionally, it facilitates replication and comparison across studies, contributing to a more robust body of evidence in the field. Researchers should also focus on the potential of instant messaging to tailor interventions based on user feedback and behavior, enhancing personalization and effectiveness. Studies could investigate the impact of instant messaging on different health behaviors and across diverse populations, particularly in reaching people who may not have access to traditional healthcare resources. Additionally, exploring the integration of instant messaging with other digital health tools, such as wearable devices, could provide comprehensive support and monitoring for users.

Moreover, further investigation into the mechanisms underlying interventions could provide deeper insights into how and why certain strategies work, informing the development of more effective digital health solutions. Collaborations across disciplines, including behavioral science, technology, and public health, will advance this field.

### 4.2 Strengths and Limitations

This is the first meta‐analysis to comprehensively evaluate the effectiveness of instant messaging applications on health behavior change. The conduct of the review followed our original protocol that is rigorous and openly available. While previous reviews only focused on a single or a few health behaviors, our review synthesizes the existing literature, encompassing as many as seven key health behaviors. This comprehensive approach provides a holistic understanding of the effectiveness of instant messaging‐based interventions on multiple health behaviors.

Still, this study has some limitations. Firstly, we only included articles published in English, potentially overlooking studies published in other languages or databases. Secondly, the results of this study are influenced by common limitations in health behavior research, such as the self‐reported nature of most outcome measurements and self‐selection bias. Thirdly, since most of the included studies were conducted in China, this may affect the generalizability of the findings to other populations. Fourthly, while our results indicate that instant messaging‐based interventions show the potential to promote health behavior change, their effectiveness may be influenced by contextual and methodological factors not fully examined in this meta‐analysis. For example, variations in intervention design, such as differences in message personalization (e.g., theory‐driven versus generic content), delivery frequency, participants' preferred timing for receiving messages, and human versus automated interactions, could account for the observed variability in outcomes. Similarly, population‐specific factors, such as cultural norms influencing dietary habits and digital literacy, may limit generalizability. Additionally, variations in outcome measures (e.g., self‐reports vs. objective biomarkers like HbA1c level) and intervention duration (short‐term vs. long‐term) may influence the findings. These unmeasured variables likely contribute to the mixed efficacy across studies and highlight the need for future research to systematically evaluate how intervention components, cultural contexts, and measurement approaches interact with effectiveness.

### 4.3 Linking Evidence to Action

- Instant messaging‐based interventions are promising in enhancing health behavior change, including physical activity, sleep, and smoking cessation.
- Leveraging real‐time communication and multimedia content can improve patient engagement and intervention effectiveness.

## 5 Conclusions

This meta‐analysis demonstrated that instant messaging‐based interventions yielded a statistically significant beneficial effect on health behavior change, including physical activity, sleep, and smoking cessation. Future research should focus on exploring the long‐term sustainability of these interventions and examining their applicability across diverse populations and settings.

## Conflicts of Interest

The authors declare no conflicts of interest.

## Supporting information


**Table S1.** Search strategy for PubMed.
**Table S2.** Summary of findings.
**Table S3.** Risk of bias assessment of the included studies (*N* = 57).
**Figure S1.** PRISMA flow diagram for systematic reviews.
**Figure S2.** Leave‐One‐Out analysis result.
**Figure S3.** Galbraith plot.
**Figure S4.** Funnel plots to assess publication bias with smoking cessation studies.

## Data Availability

The data that support the findings of this study are available from the corresponding author upon reasonable request.
