# Supplementary material for: The Effectiveness of Instant Messaging‐Based Interventions on Health Behavior Change: A Systematic Review and Meta‐Analysis
Source: Worldviews Evid Based Nurs. 2025 Jul 27;22(4):e70066. doi: 10.1111/wvn.70066 (PMC12301180; doi:10.1111/wvn.70066)
Supplement: Supplementary file 1 — Table S1. Search strategy for PubMed. Table S2. Summary of findings. Table S3. Risk of bias assessment of the included studies (N = 57). Figure S1. PRISMA flow diagram for systematic reviews. Figure S2. Leave‐One‐Out analysis result. Figure S3. Galbraith plot. Figure S4. Funnel plots to assess publication bias with smoking cessation studies. [file WVN-22-0-s001.docx]

**Supplementary Materials**

Supplementary Materials1

Table S1. Search strategy for PubMed2

Table S2. Summary of findings3

Table S3. Risk of bias assessment of the included studies (N = 57)4

Figure S1. PRISMA flow diagram for systematic reviews6

Figure S2. Leave-One-Out analysis result7

Figure S3. Galbraith plot8

Figure S4. Funnel plots to assess publication bias with smoking cessation studies9

| **SUPPLEMENTARY MATERIAL TABLE S1** Search strategy for PubMed. | |
| --- | --- |
| Items | Search terms |
|  | "exercise"[MeSH Terms] OR "physical activit*"[Title/Abstract] OR "health behav*"[Title/Abstract] OR "behavior change"[Title/Abstract] OR "behaviour change"[Title/Abstract]  "Sedentary Behavior"[MeSH Terms] OR "sedentary lifestyle"[Title/Abstract] OR "sedentar*"[Title/Abstract]  "Sleep Hygiene"[MeSH Terms] OR "insomnia"[Title/Abstract] OR "sleep"[Title/Abstract]  "diet"[MeSH Terms] OR "nutrition*"[Title/Abstract] OR "food habits"[Title/Abstract]  "early detection of cancer"[MeSH Terms] OR "cancer screening"[Title/Abstract] OR "cancer screening tests"[Title/Abstract] OR "cancer early diagnosis"[Title/Abstract]  "smoking cessation*"[MeSH Terms] OR "smoking reduction*"[MeSH Terms] OR "tobacco use cessation*"[MeSH Terms] OR "stop smoking"[Title/Abstract] OR "quit smoking"[Title/Abstract]  "alcohol drinking"[MeSH Terms] OR "alcohol intake"[Title/Abstract] OR "alcohol drinking"[Title/Abstract] OR "alcohol consumption"[Title/Abstract] |
|  | "chat-based"[Title/Abstract] OR "Facebook Messenger"[Title/Abstract] OR "WhatsApp"[Title/Abstract] OR "WeChat"[Title/Abstract] OR "Telegram"[Title/Abstract] OR "Skype"[Title/Abstract] OR "instant messaging"[Title/Abstract] OR "chat"[Title/Abstract] |
|  | "randomized controlled trial"[Title/Abstract] OR "Randomized"[Title/Abstract] OR "randomised"[Title/Abstract] OR "RCT"[Title/Abstract] |

| **SUPPLEMENTARY MATERIAL TABLE S2** Summary of findings. | | | | | | | | | | | | |
| --- | --- | --- | --- | --- | --- | --- | --- | --- | --- | --- | --- | --- |
| Certainty assessment | | | | | | | | No of participants | | Effect | Certainty |  |
| Outcome | No of studies | Study design | Risk of bias | Inconsistency | Indirectness | Imprecision | Publication bias | Treatment | Control |  |  |  |
| 6MWT | 2 | RCT | Serious | Not serious | Not serious | Serious | None | 149 | 148 | SMD: 0.96 (95%CI: 0.72, 1.20) | ⨁⨁◯◯ Low |  |
| MET | 3 | RCT | Serious | Serious | Not serious | Serious | None | 128 | 128 | SMD: 0.35 (95%CI: -0.27, 0.97) | ⨁◯◯◯ Low |  |
| MVPA | 2 | RCT | Serious | Not serious | Not serious | Not serious | None | 177 | 178 | SMD: 0.21 (95%CI: 0.00, 0.42) | ⨁⨁⨁◯  Moderate |  |
| Sedentary time | 3 | RCT | Serious | Not serious | Not serious | Not serious | None | 194 | 198 | SMD: 0.25 (95%CI: -0.24, 0.74) | ⨁⨁⨁◯  Moderate |  |
| Sleep quality | 4 | RCT | Serious | Not serious | Not serious | Not serious | None | 251 | 247 | SMD: -0.39 (95%CI: -1.44, -0.42) | ⨁⨁⨁◯  Moderate |  |
| HbA1c | 3 | RCT | Serious | Serious | Not serious | Not serious | None | 185 | 170 | SMD: -0.45 (95%CI: -1.38, 0.48) | ⨁⨁⨁◯  Moderate |  |
| BMI | 5 | RCT | Serious | Not serious | Not serious | Not serious | None | 280 | 265 | SMD: -0.10 (95%CI: -0.30, 0.10) | ⨁⨁⨁◯  Moderate |  |
| Energy intake | 2 | RCT | Serious | Serious | Not serious | Serious | None | 139 | 132 | SMD: 0.64 (95%CI: 0.01, 1.27) | ⨁◯◯◯ Low |  |
| Hemoglobin | 2 | RCT | Serious | Serious | Not serious | Serious | None | 93 | 93 | SMD: 0.32 (95%CI: -0.29, 0.93) | ⨁◯◯◯ Low |  |
| Validated abstinence rate | 13 | RCT | Serious | Not serious | Not serious | Not serious | None | 3554 | 3537 | OR: 1.88 (95% CI: 1.28, 2.74) | ⨁⨁⨁◯  Moderate |  |
| Note: 6MWT: 6-minute walk test; MET: metabolic equivalents; MVPA: moderate-to-vigorous physical activity; HbA1c: Glycated Hemoglobin; BMI: Body Mass Index; CI: Confidence Interval; OR: Odds Ratio; SMD: Standardized Mean Difference | | | | | | | | | | | |  |

| **SUPPLEMENTARY MATERIAL TABLE S3** Risk of Bias assessment of the included studies (*n* = 57). | | | | | | |
| --- | --- | --- | --- | --- | --- | --- |
| Study | Risk of Bias Domain | | | | | Overall Risk |
|  | Domain 1: | Domain 2: | Domain 3: | Domain 4: | Domain 5: |  |
|  | Randomization process | Deviations From the Intended Intervention | Missing Outcome Data | Measurement of the Outcome | Selection of the Reported Result |  |
| Alshahrani 2021 | Low | Some concerns | Low | Low | Low | Some concerns |
| Lin 2021 | Some Concerns | Low | Some concerns | Low | Low | Some concerns |
| Bi 2021 | Low | Some concerns | Some concerns | Some concerns | Low | Some concerns |
| Shokri 2024 | Low | Some concerns | Low | Low | Low | Some concerns |
| Cheung 2024 | Low | Some concerns | Low | Low | Low | Some concerns |
| Su 2021 | Low | Low | Low | Low | Low | Low |
| Xu 2020 | Low | Some concerns | Low | Low | Low | Some concerns |
| Alley 2016 | Low | Some concerns | Some concerns | Low | Some concerns | Some concerns |
| Wu 2023 | Low | Some concerns | Low | Low | Low | Some concerns |
| Kwan 2020 | Low | Some concerns | Some concerns | Low | Low | Some concerns |
| Al-Ghafri 2019 | Low | Some concerns | Low | Low | Low | Some concerns |
| Valle 2022 | Low | Some concerns | Low | Low | Some concerns | Some concerns |
| Peng 2017 | Low | Some concerns | Low | Low | Low | Some concerns |
| Chan 2022 | Low | Some concerns | Low | Low | Low | Some concerns |
| Saquib 2023 | Low | Some concerns | Low | Low | Low | Some concerns |
| Li 2023 | Some concerns | Low | Some concerns | Low | Some concerns | Some concerns |
| Klaren 2014 | Low | Low | Some concerns | Low | Low | Some concerns |
| Gieselmann 2019 | Low | Some concerns | Low | Low | Low | Some concerns |
| Han 2021 | Low | Some concerns | Low | Low | Low | Some concerns |
| Wang 2023 | Low | Some concerns | Low | Low | Low | Some concerns |
| Duan 2023 | Low | Some concerns | Low | Low | Low | Some concerns |
| Li 2022 | Low | Low | Low | Low | Low | Low |
| Ye 2024 | Low | Some concerns | Low | Low | Low | Some concerns |
| Wang 2022 | Low | Some concerns | Low | Low | Low | Some concerns |
| Al-Hamdan 2021 | Low | Some concerns | Low | Low | Low | Some concerns |
| Kang 2021 | Low | Some concerns | Low | Low | Low | Some concerns |
| Lin 2021 | Low | Some concerns | Low | Low | Low | Some concerns |
| Liu 2024 | Low | Some concerns | Some concerns | Low | Low | Some concerns |
| Chen 2023 | Low | Low | Some concerns | Low | Low | Some concerns |
| Zhang 2022 | Low | Low | Some concerns | Low | Low | Some concerns |
| Ahmad 2018 | Low | Low | Low | Low | Low | Some concerns |
| Alghafri 2018 | Low | Some concerns | Some concerns | Some concerns | Low | Some concerns |
| Ding 2020 | Low | Some concerns | Low | Low | Low | Some concerns |
| Xia 2022 | Low | Low | Some concerns | Low | Some concerns | Some concerns |
| Ghasemian 2024 | Low | Some concerns | Low | Some concerns | Low | Some concerns |
| Luo 2022 | Low | Low | Some concerns | Low | Low | Some concerns |
| Zhao 2021 | Low | Some concerns | Some concerns | Some concerns | Low | Some concerns |
| Li 2024 | Some concerns | Low | Some concerns | Low | Low | Some concerns |
| Cheung 2015 | Some concerns | Low | Some concerns | Some concerns | Some concerns | Some concerns |
| Chen 2020 | Low | Some concerns | Low | Low | Low | Some concerns |
| Durmaz 2019 | Low | Low | Some concerns | Low | Low | Some concerns |
| Lin 2022 | Low | Some concerns | Some concerns | Some concerns | Low | Some concerns |
| Luo 2021 | Low | Some concerns | Low | Low | Low | Some concerns |
| Tang 2023 | Low | Low | Some concerns | Some concerns | Low | Some concerns |
| Weng 2023 | Low | Some concerns | Low | Low | Low | Some concerns |
| Wang 2019 | Low | Low | Low | Some concerns | Low | Some concerns |
| Weng 2021 | Low | Some concerns | Low | Some concerns | Low | Some concerns |
| Balmumcu 2021 | Low | Some concerns | Some concerns | Low | Low | Some concerns |
| Guo 2023 | Low | Some concerns | Some concerns | Low | Low | Some concerns |
| Luk 2023 | Low | Some concerns | Low | Some concerns | Low | Some concerns |
| Rojnawee 2023 | Low | Some concerns | Low | Low | Low | Some concerns |
| Zhao 2024 | Low | Some concerns | Some concerns | Low | Low | Some concerns |
| Li 2022 | Low | Some concerns | Some concerns | Low | Low | Some concerns |
| Wu 2024 | Low | Some concerns | Low | Low | Low | Some concerns |
| Wang 2021 | Low | Low | Low | Low | Low | Low |
| Zhang 2021 | Low | Low | Some concerns | Some concerns | Low | Some concerns |
| Chau 2024 | Low | Some concerns | Low | Low | Low | Some concerns |

**Identification**

Title and abstract review **(n = 1084)**

Studies for eligibility **(n = 85)**

References removed **(n = 1051)**

Duplicates identified manually (n = 4)

Duplicates identified by Covidence (n = 957)

Marked as ineligible by automation tools (n = 90)

Studies excluded **(n = 999)**

Studies excluded **(n = 28)**

**Included**

Studies included in review **(n = 57)**

**Screening**

Studies from databases/registers **(n = 2135)**

ClinicalTrials.gov (n = 841)

Web of Science (n = 529)

Embase (n = 387)

PubMed (n = 261)

PsycINFO (n = 72)

CINAHL (n = 45)

**SUPPLEMENTARY MATERIAL FIGURE S1** PRISMA flow diagram for systematic reviews.


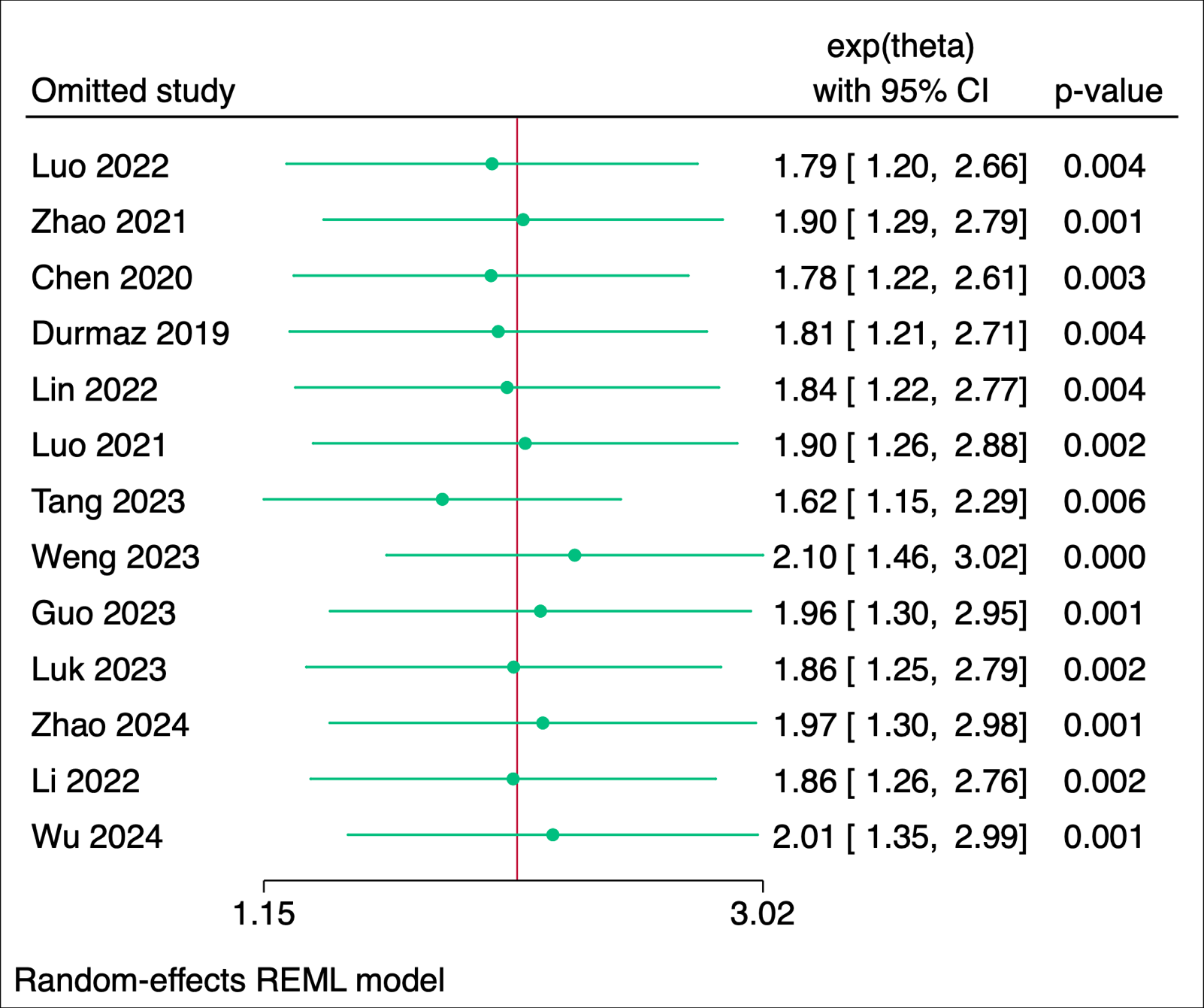


**SUPPLEMENTARY MATERIAL FIGURE S2** Leave-One-Out analysis result.


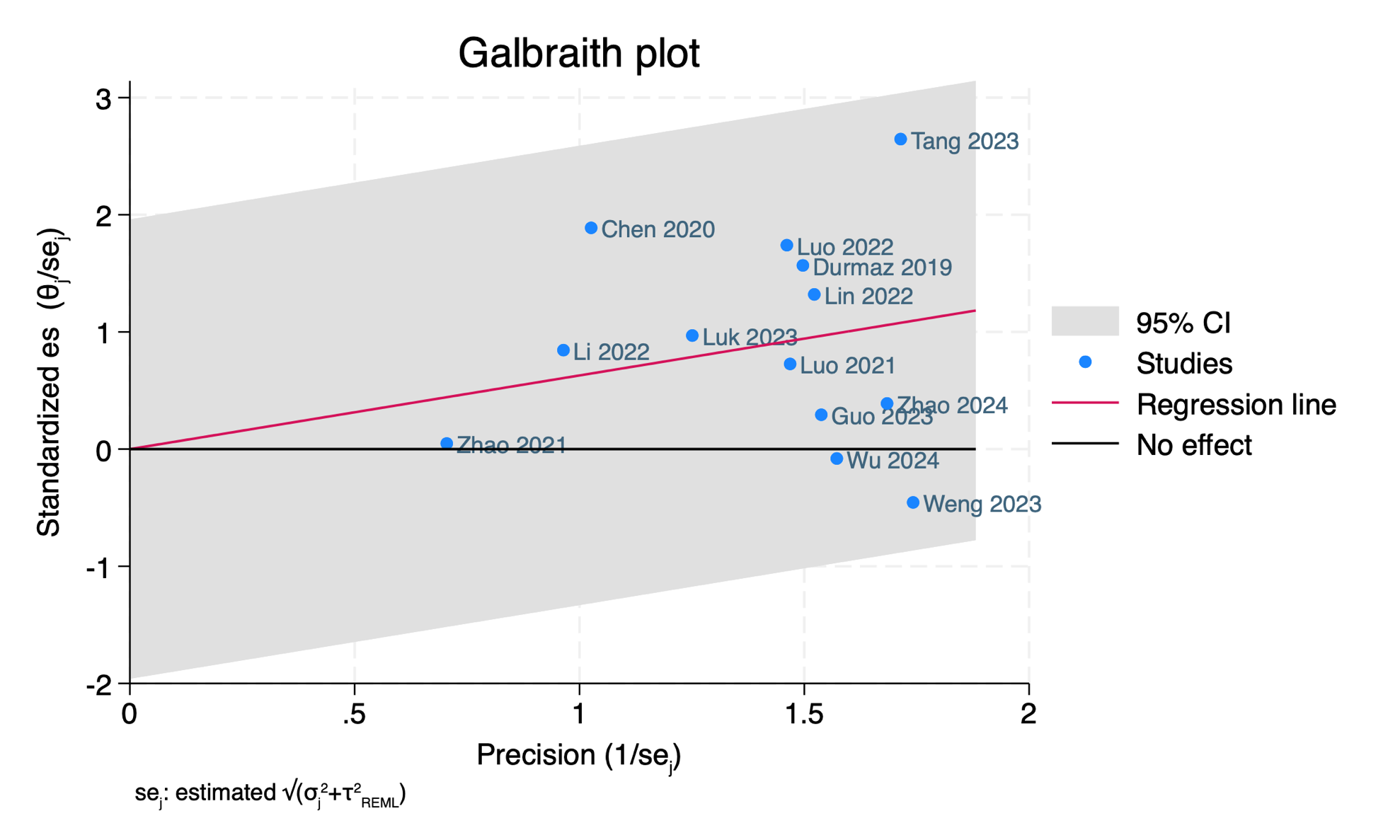


**SUPPLEMENTARY MATERIAL FIGURE S3** Galbraith plot.


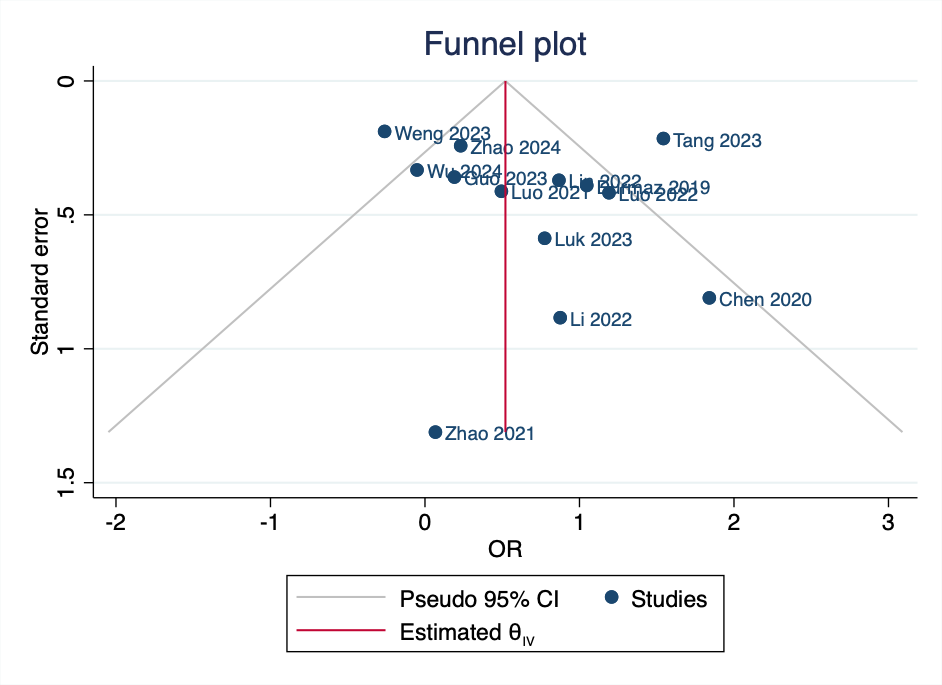


**SUPPLEMENTARY MATERIAL FIGURE S4** Funnel plots to assess publication bias with smoking cessation studies.
